# Ferroptosis and microbial pathogenesis

**DOI:** 10.1371/journal.ppat.1009298

**Published:** 2021-03-04

**Authors:** Qing Shen, Naweed I. Naqvi

**Affiliations:** Temasek Life Sciences Laboratory; and Department of Biological Sciences, National University of Singapore, Singapore; University of Maryland, Baltimore, UNITED STATES

## Introduction

Precisely controlled cell death plays a key role in development and disease in eukaryotes. Ferroptosis is a newly defined form of iron-dependent cell death best known for its role in tumor suppression in mammalian cells [1–3]. To date, ferroptosis or ferroptosis-like cell death has been observed in pathogen-challenged rice and tobacco leaves [4–6], heat-stressed Arabidopsis roots [7], and in the sleeping sickness causal parasite *Trypanosoma brucei* [8], but not in any microbial system (e.g., bacteria, archaea, or fungi) as yet. Recently, the occurrence of ferroptosis was confirmed during pathogenic development in the rice-blast fungus *Magnaporthe oryzae*, and the contribution of such regulated cell death to virulence of this rice pathogen was highlighted [5]. Collectively, these findings set forth a new area of research in microbial pathogenesis and molecular host–microbe interactions; and suggest novel strategies for pathogen control based on modulating ferroptotic death and/or iron homeostasis.

## What is ferroptosis?

Ferroptosis is a regulated mode of cell demise driven by iron-dependent peroxidation of membrane lipids [1–3,9]. Such cell death can be induced upon failure of the lipid peroxide reducing system that involves glutathione peroxidase 4 (GPX4) [10,11], which enzymatically converts phospholipid hydroperoxides to nontoxic lipid alcohols using glutathione (GSH) as a cosubstrate [12]. Accordingly, either GSH depletion caused by buthionine sulfoximine (BSO) or Erastin treatment, or GPX4 inactivation through gene deletion or pharmacological inhibition, leads to lethal accumulation of lipid peroxides in cellular membranes and results in strong induction of ferroptosis [10,11]. Such death, however, can be blocked by lipophilic antioxidants such as liproxstatin-1 (Lip-1) and ferrostatin-1 (Fer-1), which were selected from chemico-genetic screens and established as specific inhibitors of ferroptosis [10,13]. Like exogenous lipophilic antioxidants, endogenous ones such as the reduced Coenzyme Q_10_ (CoQ_10_) and Tetrahydrobiopterin (BH_4_) are capable of potently suppressing ferroptosis as membrane radical-trappers, and thus contribute to a GPX4-independent lipid peroxide detoxification mechanism [14–16].

In addition to accumulation of lethal levels of lipid peroxides, ferroptosis is characterized by its strict iron dependency as well. Ferroptotic death can be prevented through iron chelation [13], whereas iron supplementation enhances or directly activates such type of cell mortality [13,17]. Ferroptosis sensitivity is modulated by the import, storage, and export of iron [18,19]; and iron uptake via the transferrin receptor serves as a specific marker for ferroptosis [20]. Furthermore, initiation of ferroptosis is enabled by iron-dependent metabolic enzymes, such as NADPH oxidase (Nox) and lipoxygenase, that lead to peroxidation of membrane lipids [1,13]. For instance, the membrane spanning Nox enzymes have 2 iron-containing hemes, which are noncovalently bound to it and participate in the electron transfer from NADPH to O_2_ and the consequent superoxide production [21]. Superoxides generated subsequently interact with membrane lipids and trigger cell death once GPX4 and the requisite antioxidant systems are inactivated.

## What function does ferroptosis serve in *M*. *oryzae* development?

**Fig 1:**
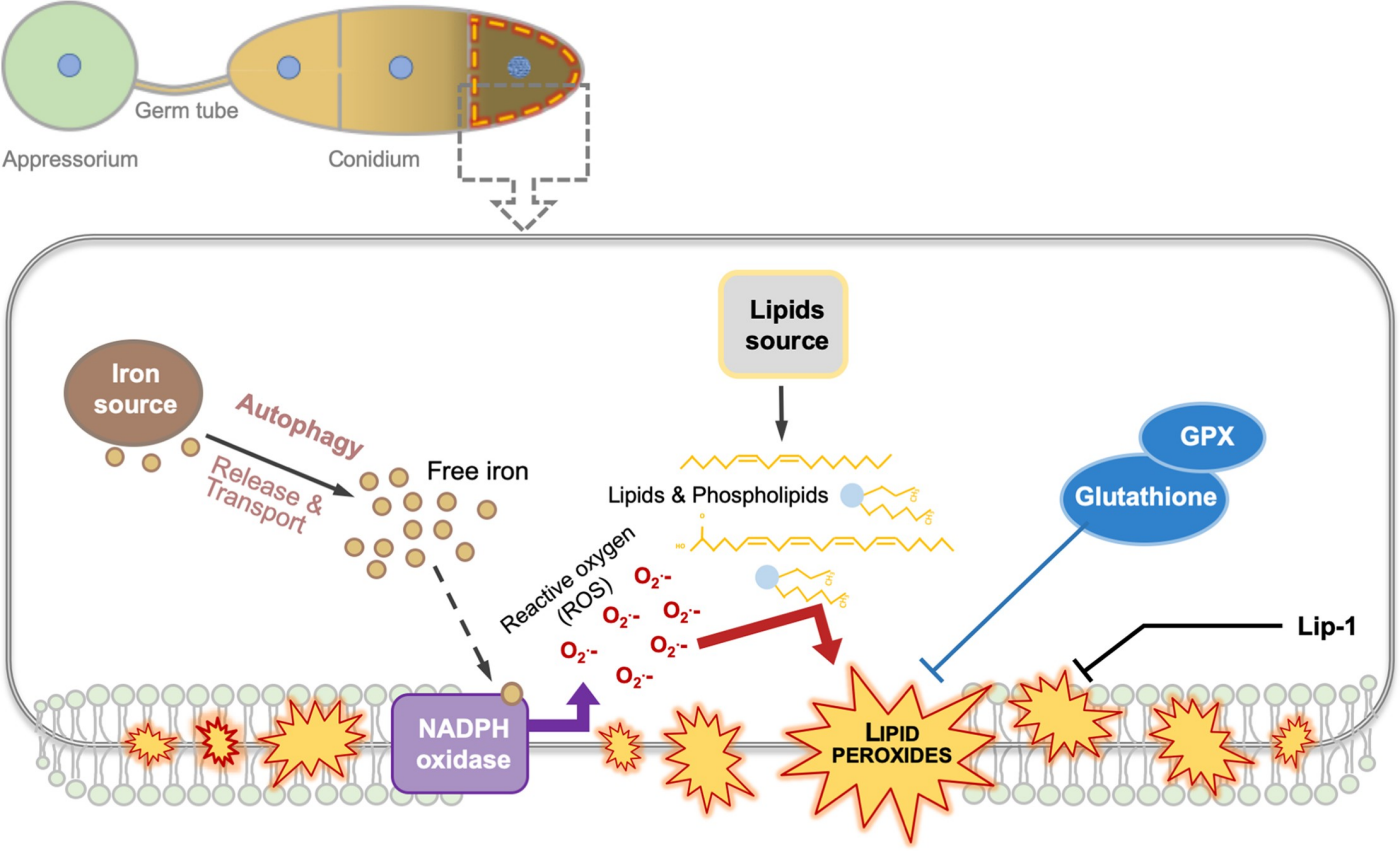
Ferroptosis occurs sequentially in the 3-celled conidium and is essential for pathogenesis in *Magnaporthe oryzae*. In *M*. *oryzae*, ferroptosis initiates first in the terminal conidial cell distal to the infection structure (appressorium). Within this cell, lipid peroxides are generated via the iron-dependent NADPH oxidase activity that accrues in the plasma membrane and trigger cell death as assessed by nuclear and cellular degradation. The reduced GSH-dependent GPX function acts as a negative regulator of such death-inducing lipid peroxides. Ferroptosis subsequently spreads to the middle and proximal conidial cells and the germ tube prior to appressorium maturation. The ferroptosis-enabling iron is acquired from intracellular source(s) in *M*. *oryzae* and is transported via autophagy, although the nature of such iron source is still unclear. The ferroptosis inhibitor Lip-1 is a lipophilic antioxidant that acts as a potent suppressor of conidial ferroptosis in rice blast. Nuclei and lipid peroxides are indicated as blue circles and orange dashes, respectively, in the conidial cell(s) undergoing ferroptosis. GPX, glutathione peroxidase; GSH, glutathione; Lip-1, liproxstatin-1; ROS, reactive oxygen species.

Ferroptosis contributes to the programmed cell death of the 3-celled asexual spores, also known as conidia, in *M*. *oryzae* (Fig 1) [5], which causes blast disease in several important cereal crops such as rice and wheat, and adversely impacts global agriculture [22]. Pathogenic life cycle of *M*. *oryzae* starts when the 3-celled conidium germinates on the leaf surface, and produces a polarized germ tube, which forms a specialized infection structure called the appressorium, which breaches the leaf epidermis using enormous turgor and a thin but rigid penetration peg [22]. Bulbous fungal hyphae differentiated from penetration pegs then resume filamentous growth within the plant cells and finally kill them and produce more conidia using the host-derived nutrients [22]. During appressorium maturation, the 3 conidial cells transport their cellular contents to the developing appressorium and then degrade their nuclei and undergo a specific autophagic cell death [22,23].

Such programmed death can be suppressed by iron chelators or the ferroptosis inhibitor Lip-1, and this death suppression is invariably accompanied by a dramatic decrease in lipid peroxide levels in the plasma membrane [5]. Lack of Nox activity through genetic or pharmacological inhibition simulates iron chelation or Lip-1–based inhibition in terms of suppressing conidial death and the associated lipid peroxidation [5]. Conversely, iron supplementation or GSH depletion via BSO drives peroxidation of membrane lipids and advances conidial ferroptosis [5]. These typical characteristics confirmed the occurrence of ferroptosis in the 3 conidial cells in a highly controlled and sequential manner: Ferroptosis initiates first in the terminal conidial cell distal to the appressorium, and then sequentially spreads to the middle and proximal cells (Fig 1) [5]. Such precise execution of conidial death may be attributable to the wave-like nature of ferroptosis propagation [24]. In mammalian cell populations or tissues, ferroptosis spreads as a wave in response to iron supplementation or GSH depletion, but not GPX4 inactivation [24,25]. Such unique propagation suggests a cell–cell communication that delivers ferroptosis trigger(s), which is supported by the dynamic spread of conidial ferroptosis in *M*. *oryzae* too. The abundance of iron increases considerably within the terminal conidial cell before it undergoes ferroptosis [5]. Such iron accumulation followed by cell death appears subsequently in the middle and proximal cells following the same chronology or sequence of conidial death [5], thus implying iron as a propagation trigger that fine-tunes and controls the crucial conidial ferroptosis in rice blast.

## What is the role of ferroptosis/iron homeostasis in microbial pathogenesis?

Regarding this question, what is known is that ferroptotic conidial death during appressorium development determines proper pathogenesis of *M*. *oryzae* [5]. When ferroptosis is subverted in the conidium through iron chelation or Lip-1–based inhibition or Nox inactivation, *M*. *oryzae* is unable to colonize rice cells and fails to cause the typical blast disease lesions. In contrast, an additional supply of iron boosts the conidial cell death and also increases the ability of *M*. *oryzae* to infect the rice plants.

So how does conidial ferroptosis impact the infection ability of *M*. *oryzae*? One possibility could be that successful ferroptosis occurs within a limited time period/window, when the nutrients stored in the conidium can still support its life activities, and guarantees proper development of the infection structure/appressorium. This is supported by the observation that smaller or immature appressoria (unable to penetrate the host by implication) are inevitably produced upon disruption of conidial ferroptosis [5]. Further investigation is required to unveil the link between conidial ferroptosis and the proper morphogenesis or formation of a functional appressorium.

To date, it is unclear whether ferroptosis occurs in microbial pathogens other than *M*. *oryzae*. However, the ability to take up iron and maintain iron homeostasis is essential for full virulence of an array of microbial pathogens, with hosts ranging from plants to humans [26,27]. For example, the human pathogenic fungus *Candida albicans* employs a high-affinity iron permease system [28] and utilizes siderophores produced by other microbes for iron acquisition [29–31]. *C*. *albicans* also uses a series of transporters that deliver heme-iron across the cell wall, and then takes it up through endocytosis, to ensure proper iron uptake during growth and colonization within the host, thus enabling a strong infection capability [31]. In line with the iron requirement, Nox function and lipoxygenase activities, which are sources of lipid peroxidation in mammals, have been reported in fungi and bacteria too [32–34]. Thus, it will be interesting to investigate whether iron-dependent ferroptosis serves as an evolutionary conserved mechanism widely involved in microbial pathogenesis.

## What is the source of iron in ferroptosis?

The availability of iron from external host-derived sources or growth medium is extremely limited during pathogenic differentiation prior to host penetration in *M*. *oryzae*. As such, it is most likely the internally stored iron that enables and supports ferroptosis in the rice blast fungus. Presently, the nature of such internal source(s) and the type of iron involved is unclear. In mammalian cells, internal iron is stored as ferric ion in the form of ferritin complexes, and a selective form of autophagy referred to as ferritinophagy is responsible for ferritin degradation, thus releasing free iron for ferroptosis [35]. Although, *M*. *oryzae* lacks ferritin-like complexes, autophagy is still involved in fine-tuning ferroptosis likely through the trafficking and/or distribution of iron (Fig 1) [5]. It remains to be seen whether such regulation of intracellular ferric ions is indicative of the potential intrinsic stores (endoplasmic reticulum, mitochondria, and/or vacuoles) and sinks for iron in microbial pathogens that affect plants and animals. A key issue that needs to be resolved in the near future is what triggers and executes the release of ferroptosis-enabling iron in the pathogen. Lastly, it would be interesting to address whether the host plays a role in directly or indirectly regulating ferroptosis in the microbial pathogen, for instance, by modulating iron availability at the crucial stages of pathogenic development therein.
